# Arctic warming by abundant fine sea salt aerosols from blowing snow

**DOI:** 10.1038/s41561-023-01254-8

**Published:** 2023-09-04

**Authors:** Xianda Gong, Jiaoshi Zhang, Betty Croft, Xin Yang, Markus M. Frey, Nora Bergner, Rachel Y.-W. Chang, Jessie M. Creamean, Chongai Kuang, Randall V. Martin, Ananth Ranjithkumar, Arthur J. Sedlacek, Janek Uin, Sascha Willmes, Maria A. Zawadowicz, Jeffrey R. Pierce, Matthew D. Shupe, Julia Schmale, Jian Wang

**Affiliations:** 1 Center for Aerosol Science and Engineering, Department of Energy, Environmental and Chemical Engineering, Washington University in St. Louis, St. Louis, MO USA; 2 Department of Physics and Atmospheric Science, Dalhousie University, Halifax, Nova Scotia Canada; 3 British Antarctic Survey, Natural Environment Research Council, Cambridge, UK; 4 Extreme Environments Research Laboratory, École Polytechnique Fédérale de Lausanne (EPFL), Sion, Switzerland; 5 Department of Atmospheric Science, Colorado State University, Fort Collins, CO USA; 6 Environmental and Climate Science Department, Brookhaven National Laboratory, Upton, NY USA; 7 Department of Environmental Meteorology, Trier University, Trier, Germany; 8 Cooperative Institute for Research in Environmental Sciences, University of Colorado Boulder, Boulder, CO USA; 9 Physical Sciences Laboratory, NOAA, Boulder, CO USA

**Keywords:** Atmospheric chemistry, Cryospheric science, Environmental impact, Atmospheric science

## Abstract

The Arctic warms nearly four times faster than the global average, and aerosols play an increasingly important role in Arctic climate change. In the Arctic, sea salt is a major aerosol component in terms of mass concentration during winter and spring. However, the mechanisms of sea salt aerosol production remain unclear. Sea salt aerosols are typically thought to be relatively large in size but low in number concentration, implying that their influence on cloud condensation nuclei population and cloud properties is generally minor. Here we present observational evidence of abundant sea salt aerosol production from blowing snow in the central Arctic. Blowing snow was observed more than 20% of the time from November to April. The sublimation of blowing snow generates high concentrations of fine-mode sea salt aerosol (diameter below 300 nm), enhancing cloud condensation nuclei concentrations up to tenfold above background levels. Using a global chemical transport model, we estimate that from November to April north of 70° N, sea salt aerosol produced from blowing snow accounts for about 27.6% of the total particle number, and the sea salt aerosol increases the longwave emissivity of clouds, leading to a calculated surface warming of +2.30 W m^−2^ under cloudy sky conditions.

## Abstract (web summary)

Fine sea salt aerosols produced by blowing snow in the Arctic impact cloud properties and warm the surface, according to observations from the MOSAiC expedition.

## Main

The climate in the Arctic has received close attention because its near-surface air temperature is increasing nearly four times faster than the global average^1^. This ‘Arctic amplification’ is a prominent and complex feature of climate change with strong impacts on human and natural systems, not only within the Arctic but also globally^2^. Aerosols play an important role in the Arctic climate by scattering and absorbing solar radiation (direct radiative effects) and by modifying the properties of clouds (indirect effects). The indirect aerosol effects in the Arctic can be very impactful because low-level clouds, containing both liquid and ice water, are highly susceptible to changes in aerosol concentration, especially when the aerosol population is limited. In addition, the sensitivity of the surface energy budget to cloud variability is high^3^. During the winter months in the Arctic, when solar radiation is mostly absent, low-level clouds warm the surface by absorption and re-emission of longwave radiation^4^. The elevated aerosol concentration due to Arctic haze has been shown to increase cloud droplet number concentration (CDNC) and longwave emissivity, resulting in an estimated surface warming under cloudy skies of between +3.3 and +5.2 W m^−2^ or 1 and 1.6 °C (ref. ^5^). An increase in aerosol concentration also yields smaller cloud droplets, which are expected to inhibit the formation of drizzle/rain and ice precipitation, leading to enhanced cloudiness (that is, higher liquid water path (LWP) and cloud coverage) and additional surface warming in the central Arctic^6^.

Arctic clouds radiatively warm the surface throughout the year except for a period of surface cooling in the middle of summer^7^. However, the overall effects of aerosols on Arctic clouds and climate remain unclear^8^. This uncertainty is, to a large degree, due to the poor understanding of aerosol sources and properties in the central Arctic, which prevents us from representing them adequately in numerical models^8^. During summer and early fall, Arctic aerosol is dominated by local emissions, as the Arctic front is located further north and the polar dome inhibits the transport of pollution from mid-latitudes^9^. Major aerosol sources during the winter and spring include both long-range transport and wind-driven local production^10,11^. Arctic haze, a winter and spring phenomenon of long-range transport of lower-latitude emissions^5,12^, can strongly increase aerosol loadings and concentrations of cloud condensation nuclei (CCN)^13,14^, which are particles that can form cloud droplets. Sea salt represents the highest mass fraction among all aerosol species during winter and spring in the Arctic^14–16^. At present, the mechanisms for the production of sea salt aerosol (SSA) are unclear^15,17,18^. Many studies attribute the SSA in the Arctic primarily to particle production by wave breaking and bubble bursting over the open ocean and leads^15,18^. However, recent model studies indicate that wintertime and springtime peaks of sea salt mass concentration in the Arctic can be successfully reproduced^17,19^ only with the inclusion of SSA production from blowing snow^20^. Field observations of sea salt mass concentration and number size distribution (from 400 nm to 10 μm in diameter) and airborne snow particles^21^ also suggest that blowing snow is a major source of SSA mass in the Antarctic during winter and early spring. Whereas SSAs can contribute to Aitken mode aerosols^22,23^, their sizes are relatively large, and number concentrations are often lower compared to aerosols from other sources^24,25^. Therefore, the conventional thinking is that while SSAs often contribute substantially to or even dominate high-latitude aerosol mass concentration^14,15^ and direct radiative effect^26^, their influences on CCN concentration (*N*_CCN_) and cloud properties are less pronounced^25,26^. A recent modelling study in the Antarctic suggests blowing-snow sublimation may generate a substantial amount of fine-mode particles^27^, and elevated concentrations of fine-mode particles during blowing-snow events at a coastal Alaskan Arctic site are reported in a recent observational study^28^. Both studies raise the possibility that blowing-snow-produced SSA may strongly affect the Arctic climate by impacting the CCN population and the properties of clouds.

To elucidate the sources and climate effects of SSA in the Arctic, we carried out comprehensive measurements of aerosols, blowing snow, clouds and meteorological parameters in the central Arctic over an entire year from September 2019 to October 2020 during the Multidisciplinary drifting Observatory for the Study of Arctic Climate (MOSAiC) expedition (Methods). We provide observational evidence that the production of fine-mode SSA from sublimating blowing snow strongly enhances central Arctic *N*_CCN_, leading to substantial surface warming in the Arctic during winter and spring.

## Sea salt aerosols from blowing snow

**Fig. 1:**
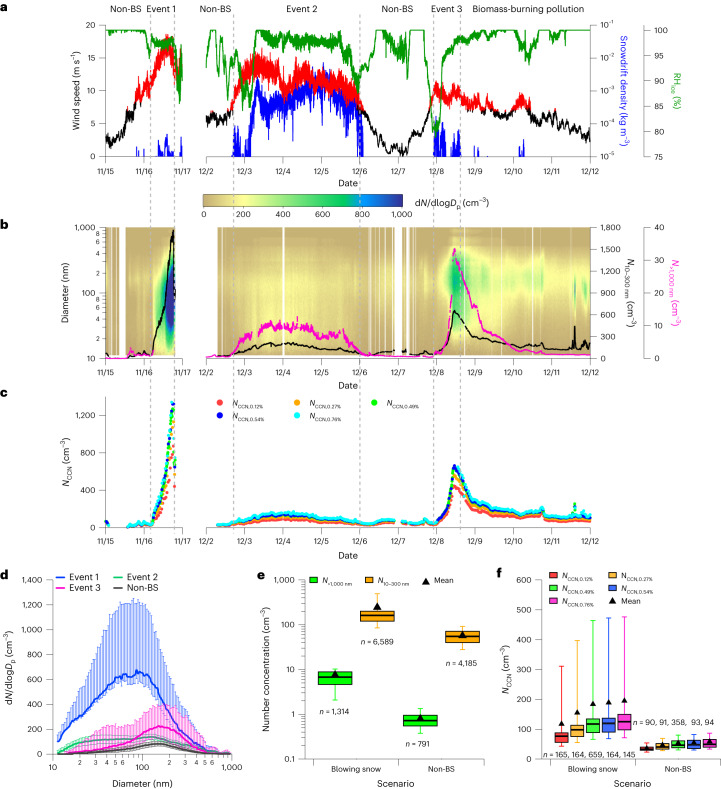
Meteorological parameters, particle size distribution and *N*_CCN_. **a**, Time series of snowdrift density at 10 m (November) and 0.1 m (December) in blue, relative humidity with respect to ice (RH_ice_) at 10 m in green and wind speed at 10 m in red (values above the threshold for blowing snow) and black (below the threshold). **b**, Contour plot of particle number size distribution (d*N*/dlog*D*_p_; here *D*_p_ represents the particle diameter) from 10 to 1,000 nm, with time series of fine-mode particle number concentration (*N*_10–__300nm_) in black and super-micron particle number concentration (*N*_>1,000nm_) in magenta. **c**, Time series of *N*_CCN_ at different supersaturations. The gray dashed lines indicate the time periods of different scenarios. **d**, Particle number size distribution during blowing-snow events and adjacent non-blowing-snow (non-BS) periods (from 00:00 on 15 to 04:00 on 16 November, 00:00 to 17:00 on 2 December and 00:00 on 6 to 22:00 on 7 December). Lines represent median values and error bars show 25th and 75th percentiles. **e**,**f**, Box plot of *N*_>1,000nm_, *N*_10–300nm_ and *N*_CCN_ at different supersaturations during blowing-snow events and non-BS periods. Centre lines, box limits and whiskers represent median values, 25th to 75th and 10th to 90th percentiles, respectively. Triangles represent mean values. The sample size used to derive the box plot is shown in the text.

**Fig. 2:**
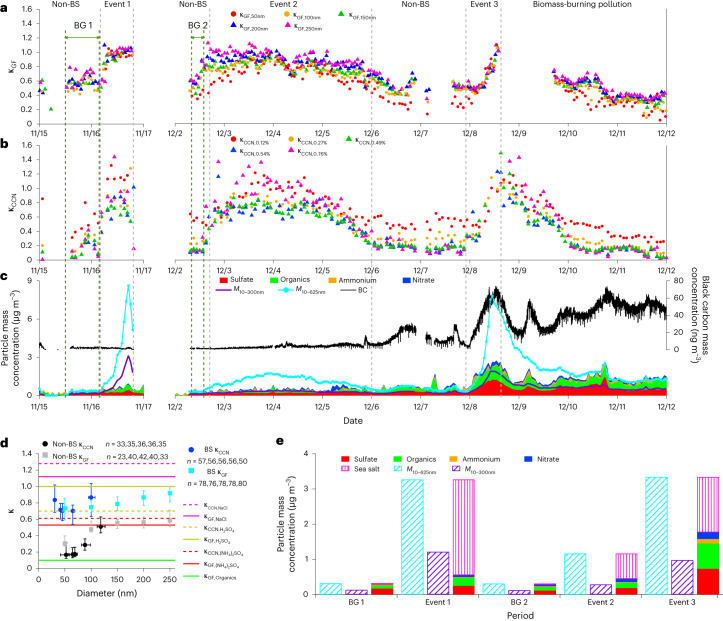
Aerosol particle hygroscopicity and chemical composition. **a**,**b**, Time series of the hygroscopicity parameters under sub-saturation derived from growth factor (κ_GF_) and supersaturations derived from cloud condensation nuclei activation (κ_CCN_). **c**, Time series of mass concentrations of non-refractory components (sulfate, organics, ammonium and nitrate) measured by ACSM, BC mass concentration (black line) and derived mass concentrations in the size ranges of 10 to 300 nm (*M*_10–300nm_) and 10 to 625 nm (*M*_10–__625nm_) in purple and cyan lines, respectively. **d**, Particle hygroscopicity as a function of particle diameter during blowing-snow (BS) events in blue (κ_CCN_) and cyan (κ_GF_) and non-blowing-snow (non-BS) periods in black (κ_CCN_) and grey (κ_GF_). Markers and error bars represent the median values and 25th to 75th percentiles. The sample size used to derive statistics is shown in the text. Particle hygroscopicities of representative species are shown as reference lines^31,32^. **e**, Bar plots of derived mass concentrations (*M*_10–__300nm_ and *M*_10–__625nm_) and mass concentrations of major species during BS events and background (BG) periods.

For this study, the blowing-snow events are identified when snowdrift density, defined as the airborne snow mass per volume of air at ambient conditions, is above 10^−5^ kg m^−3^ and wind speed exceeds the blowing-snow threshold^29^ (criteria in Methods). Measurements during three representative blowing-snow events are shown in Figs. 1 and 2. These three events occurred from 04:00 to 19:00 (all times given in UTC) on 16 November (event 1), 17:00 on 2 December to 00:00 on 6 December (event 2) and 22:00 on 7 December to 15:00 on 8 December (event 3), with average snowdrift densities representing 99, 90 and 18 percentiles of event-mean values observed at 10 m (event 1) or 0.1 m (events 2 and 3) during the MOSAiC expedition (Methods). The relative humidity with respect to ice (RH_ice_) measured at 10 m is often below 100%, facilitating the sublimation of blowing snow (Fig. 1a). Both particle number concentrations in the fine mode (10 to 300 nm in diameter, *N*_10–__300nm_) and *N*_CCN_ at five supersaturations ranging from 0.12% to 0.76% show strong increases during the blowing-snow events compared to background periods (Fig. 1b,c). For example, *N*_CCN_ during the event on 16 November is more than tenfold above that during the adjacent non-blowing-snow period. The third blowing snow event on 8 December coincides with long-range transport of biomass-burning plumes^30^. To isolate the impact of blowing snow on the aerosol population during event 3, we subtract the contribution from biomass-burning aerosol before statistical analysis (Supplementary Discussion 1). Particle number size distributions, particle number concentrations and *N*_CCN_ during blowing-snow events and non-blowing-snow periods are statistically compared in Fig. 1d–f. *N*_10–__300nm_, *N*_CCN_ and the concentration of super-micron particles (*N*_>1,000nm_) are all strongly enhanced during the blowing-snow events compared to non-blowing-snow values. On average, *N*_CCN_ increases by a factor of two to three during blowing-snow events, suggesting a potentially substantial impact on cloud properties.

Understanding the source of the fine-mode aerosols during the blowing-snow events requires knowledge of their composition. However, it is very challenging to directly measure the chemical composition of the fine-mode aerosols given their extremely low mass concentration. Here we infer size-resolved chemical composition from particle hygroscopicity measurements by taking advantage of the differences in hygroscopicity parameter (κ)^31^ among major aerosol species, including organics (κ_Organics_ ≈ 0.10), ammonium sulfate $({\kappa }_{{({{\rm{NH}}}_{4})}_{2}{{\rm{SO}}}_{4}}=0.53-0.61)$, sea salt (κ_NaCl_ = 1.12–1.28) and acidic sulfate (for example, ${\kappa }_{{{\rm{H}}}_{2}{{\rm{SO}}}_{4}}=0.70-1.00$)^31,32^. Figure 2a,b shows the time series of particle hygroscopicities under sub-saturated conditions (κ_GF_) derived from particle hygroscopic growth and under super-saturated conditions (κ_CCN_) derived from CCN activation. During the blowing-snow events, both κ_GF_ and κ_CCN_ increase to ~0.70–1.2 from non-blowing-snow values of ~0.2–0.5. Figure 2d shows the median κ values during the blowing-snow events as a function of particle diameter ranging from ~20 to 250 nm and those during non-blowing-snow periods. The elevated κ values above ~0.70 across the size range indicate the fine-mode aerosol composition during the blowing-snow events is dominated by highly hygroscopic species, that is, sea salt and/or acidic sulfate (for example, sulfuric acid).

The chemical composition of the fine-mode aerosols is further constrained by combining the time series of sulfate, organics, ammonium and nitrate mass concentrations measured by an Aerosol Chemical Speciation Monitor (ACSM) with the particle size distribution. As SSA is refractory and cannot be reliably quantified by ACSM, the total mass concentration of submicron particles is derived by integrating particle volume size distribution from 10 to 625 nm (*M*_10–625nm_). The upper size limit of 625 nm is chosen to match the vacuum aerodynamic particle diameter of 1 μm by assuming a density of 1.6 g cm^−3^ and spherical particles (Supplementary Discussion 2). The mass concentration of sea salt is then calculated as the difference between *M*_10–__625nm_ and the non-refractory submicron mass concentration measured by the ACSM. We note that the sea salt mass concentration derived using this approach could also include the contribution of refractory primary marine organics. During the non-blowing-snow periods, particle κ_CCN_ and κ_GF_ values are between 0.20 to 0.50, consistent with a minor contribution from sea salt and the dominance of submicron aerosol composition by organics and sulfate (Fig. 2e). The black carbon (BC) mass concentration remained constant and low during the first and second blowing-snow events, excluding the possibility of substantial impact by long-range-transported pollution. During these two events, sulfate mass concentration shows modest increases compared to the respective background periods (that is, increases of 45% and 63%, respectively), while the mass concentrations of fine-mode particles (*M*_10–__300nm_, derived from particle size distribution) increase much more strongly, by 780% and 130%, respectively. In addition, sulfate mass concentration during the first and second blowing-snow events can explain only 20% and 64% of the fine-mode particle mass, respectively, even if all sulfate resides in the fine mode. The minor contribution of sulfate to the increased *M*_10–__300nm_, together with the high particle hygroscopicity shown earlier, indicates that the fine-mode aerosols during the blowing-snow events are dominated by sea salt. In the absence of long-range transported pollution, the sudden emergence of a high concentration of small acidic sulfate particles is very unlikely, as acidic sulfate particles are not expected to be produced locally during the polar night. During the third blowing-snow event, aerosol was influenced by long-range transported biomass-burning plumes^30^, as indicated by the elevated BC concentration (Fig. 2c). On average, sea salt represents 47% of the submicron aerosol mass, and the fraction reached 66% from 10:00 to 15:00 on 8 December, consistent with the conclusion above that sea salt dominates the fine-mode aerosol. Particle hygroscopicity is also derived from bulk submicron aerosol composition (including sodium chloride, sulfate, organics, ammonium and nitrate) and agrees with κ_GF_ measured at all five sizes, lending additional support to the dominance of SSA during the blowing-snow events (Supplementary Fig. 1).

**Extended Data Fig. 1:**
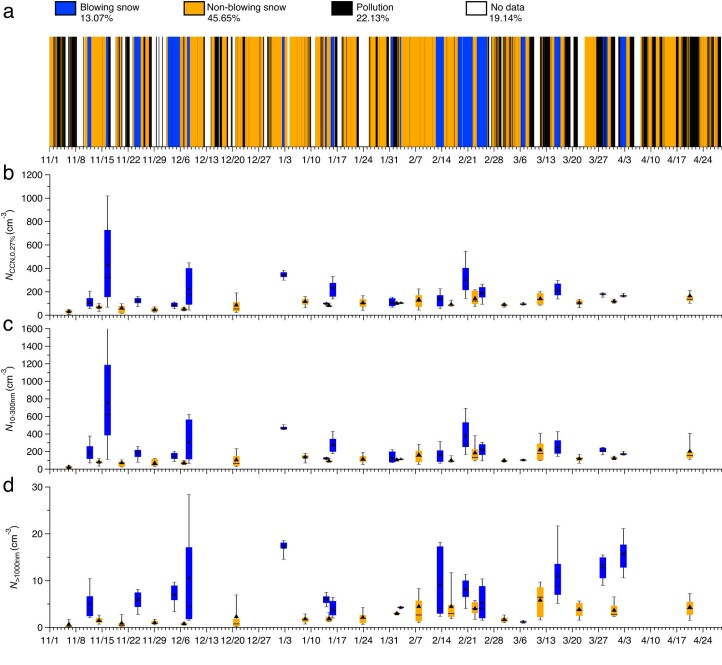
Aerosol concentrations during blowing snow events and non-blowing snow periods. **a**, The classification of periods, including blowing snow events with valid aerosol data (Blowing snow), non-blowing snow periods with valid aerosol data (Non-blowing snow), local primary pollution periods (Pollution)^60^, and periods with missing aerosol data (No data), shown as blue, orange, black, and white bars, respectively. **b-d**, Boxplot plots of CCN number concentration at supersaturation of 0.27% (*N*_CCN,0.27%_), particle number concentration in the size from 10 to 300 nm (*N*_10-300nm_), and super-micron particle number concentration (*N*_>1000nm_) during the blowing snow events (blue) and periods between two consecutive blowing snow events (orange). Center lines, box limits, and whiskers represent median values, 25^th^ to 75^th^, and 10^th^ to 90^th^ percentiles, respectively. Triangles represent mean values. The sample size used to derive the boxplot is shown in Supplementary Table 1.

We identify 29 blowing-snow events and find the events occurred over 20% of the time from November 2019 to April 2020 (Extended Data Fig. 1 and Extended Data Table 1). During these events, *N*_10–__300nm_, *N*_>1,000nm_ and *N*_CCN_ increase up to tenfold compared to periods when blowing snow is absent (Fig. 1b,c and Extended Data Fig. 1). While the results presented above indicate that the high concentrations of fine-mode aerosols are dominated by SSAs during the blowing-snow events, some of the SSAs could be produced from frost flowers and open leads under strong wind conditions. However, laboratory^33,34^ and model^17^ studies suggest that frost flowers have a minor contribution to SSA. Unlike blowing-snow events that are episodic, open leads are probably omnipresent in the central Arctic. The average open lead fraction along the trajectory of air mass arriving at the MOSAiC location shows very different temporal variations with wind speed (Supplementary Fig. 2). The observed *N*_10–__300nm_ does not correlate with the calculated emissions flux from open leads along the trajectory (Supplementary Discussion 3). While sea-spray aerosols generated from the open leads probably contribute to fine-mode particles, the lack of correlation between the open leads emissions flux and *N*_10–__300nm_ (Supplementary Fig. 3), together with the coincidence of high *N*_10–__300nm_ with elevated snowdrift density, indicates that the sublimation of blowing snow is the major source of the observed fine-mode particles.

## Mechanism of SSA production

**Fig. 3:**
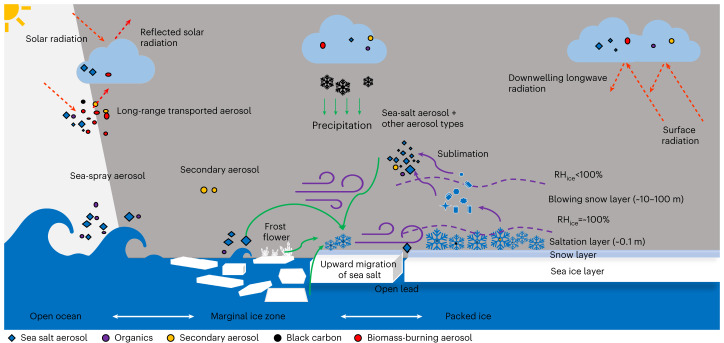
Mechanism of SSA production from the sublimation of wind-blown saline snow particles and climate impacts in the Arctic. The Arctic Ocean surface transitions from open water to the marginal ice zone and then to packed ice as surface temperature decreases. A saline snow layer overlies the sea ice (green text and lines indicate the pathway of supplying sea salt ions to marine snow). In the saltation layer, the snowdrift is transported upwards in ambient air by winds. The snow particles are lofted into the atmosphere when wind speed exceeds a critical threshold. Sublimation of blowing snow produces residual aerosol particles, including mainly sea salt (purple text and lines indicate the pathway of particle production). Together with other aerosol particles, these blowing-snow-produced SSAs both directly reflect solar radiation (direct radiative effects) and act as CCN thereby influencing cloud formation and microphysical properties (indirect radiative effects).

The mechanisms of SSA production from blowing saline snow and its impact on the Arctic climate are illustrated in Fig. 3. Previous studies suggested that snow particles are contaminated by sea water ions through several pathways, including (1) upward migration of brine from the sea ice surface into the snowpack and (2) dry and/or wet deposition of SSAs generated earlier, including wind-blown sea-spray aerosols from the open ocean, leads or polynyas and wind-blown frost flowers^20,35^. When the 10 m wind speed exceeds a critical value that ranges from 7 to 9.5 m s^−1^ under typical conditions, snow particles start saltating and get lofted into the atmosphere, reaching altitudes of several tens of metres and evolving from a drifting- to a blowing-snow event as wind speed increases^20,21,27,36^. The observation of elevated snowdrift density near the surface during MOSAiC coincides with wind speed above the critical value from ref. ^29^ (Supplementary Fig. 2). Ice sublimation reduces the size of snow particles and eventually produces residual particles consisting of all impurities contained in the snow including mainly sea salt. The size and mass concentration of SSAs are expected to be controlled by the blowing-snow particle size distribution, snow salinity, the number of sea salt particles produced per snow particle and the sublimation flux^21,27^. Elevated *N*_>1,000nm_ was observed during blowing-snow events (Extended Data Fig. 1), in agreement with the previous suggestion that blowing snow may produce an equal or higher amount of super-micron particles than the open ocean^21,27^. The fine-mode particle concentration during blowing-snow events varies from a few hundred to more than 1,000 cm^−3^, which is partly due to the large variation of snowdrift density at 0.1 m (that is, 10^−5^ to 10^−2^ kg m^−3^). The blowing-snow-produced SSA shows a broad unimodal distribution, varying from 10 to 1,000 nm, with a peak around a few tens to 100 nm. Compared to sea-spray particles generated from the open ocean/leads through bubble bursting^23,25^, sublimating blowing snow produces a relatively larger fraction of fine SSA, which is higher in number concentration, leading to a strong impact on the CCN population and thus indirect radiative effects in the Arctic as shown in the next section.

## Impact on aerosol and surface warming

**Fig. 4:**
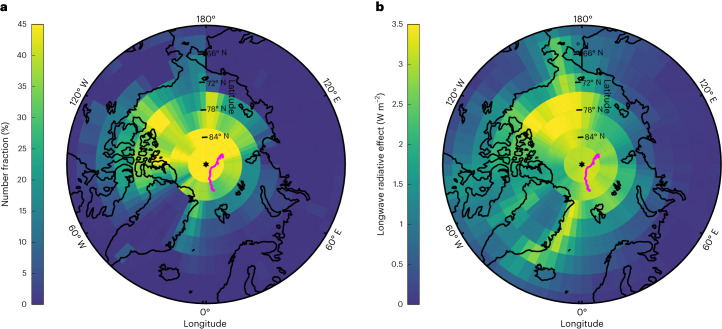
Strong SSA production from blowing snow and its climate effects in the central Arctic. **a**, GEOS-Chem-TOMAS-simulated percentage contribution of blowing-snow-produced SSA to total particle number concentration, mean values over November to April. **b**, Simulated cloud downwelling longwave radiative forcing attributed to blowing-snow-produced SSA, mean values over November to April. The magenta lines show the MOSAiC expedition track from November 2019 to April 2020.

**Extended Data Fig. 2:**
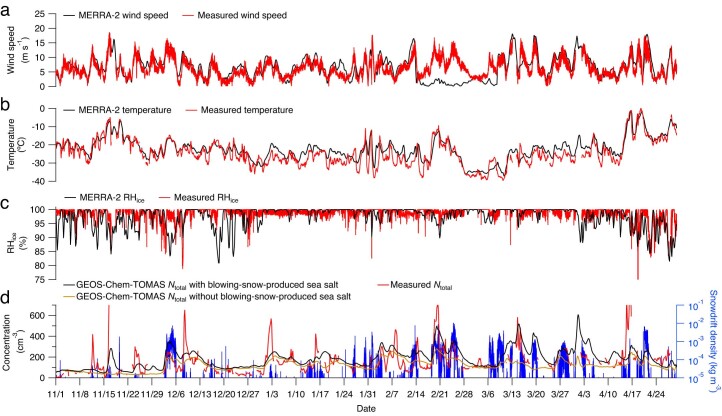
Comparison between model simulations and observations. **a-c**, Time series of wind speed, temperature, and RH_ice_ from MERRA-2 in black and measurement in red. **d**, Time series of GEOS-Chem-TOMAS model simulated total particle number concentration with blowing-snow-produced SSA included (black line) and excluded (orange line). Measured total particle number concentration and snowdrift density are shown in red and blue lines, respectively.

**Extended Data Fig. 3:**
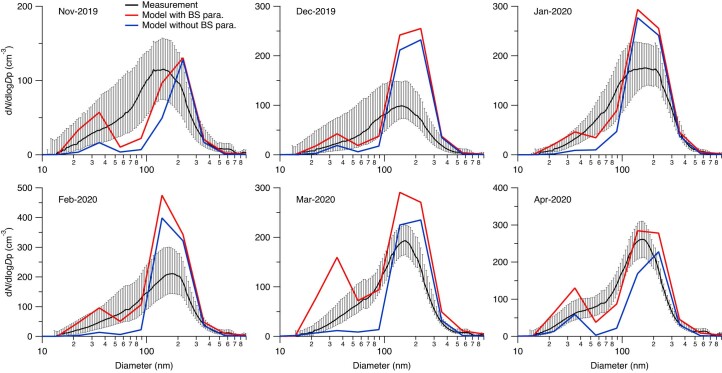
Comparison between model-simulated (NP = 5, base simulation) and measured submicron particle number size distribution. The monthly median values of the measured particle number size distribution are shown in black lines, with error bars showing the 25^th^ to 75^th^ percentiles. The monthly median values of particle number size distribution from the base simulation (NP = 5) with and without blowing-snow-produced SSA included are shown in red and blue lines, respectively.

We implement a blowing-snow aerosol emission scheme^20,27^ in the GEOS-Chem-TOMAS global chemical transport model^17^, which is used to simulate the number concentrations of blowing-snow-produced SSAs in the Arctic from November 2019 to April 2020 (Methods). The model simulation successfully captures the increase of total particle number concentration (Methods and Extended Data Fig. 2) at the MOSAiC location during the blowing-snow events. Including blowing-snow-produced SSA also better reproduces the measured particle size distribution (Extended Data Fig. 3). Averaged over the entire period from November to April in the region north of 70° N, blowing-snow-produced SSA represents about 27.6% of the total particle number (Fig. 4a).

**Extended Data Fig. 4:**
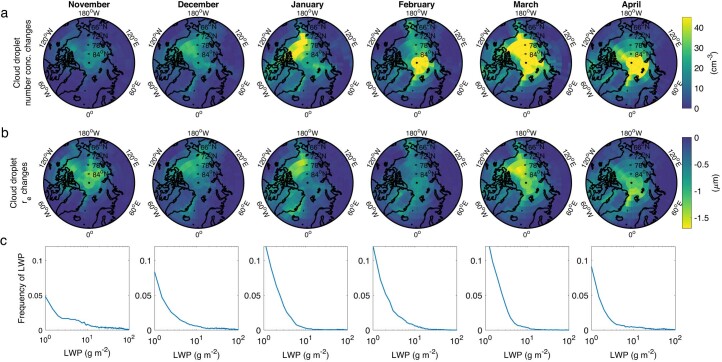
Impact of blowing-snow-produced particles on cloud properties simulated by GEOS-Chem-TOMAS and measured liquid water path. **a**, Monthly mean of the change in boundary layer cloud droplet number concentration due to blowing-snow-produced SSA from November to April. **b**, Change in boundary layer cloud effective radius (${r}_{e}$) due to blowing-snow-produced SSA estimated from the monthly mean droplet number concentration change. **c**, The frequency distribution of measured liquid water path (LWP) under cloudy skies from November to April.

The measured LWP at the MOSAiC location is mostly below 40 g m^−2^ from November to April (Extended Data Fig. 4), suggesting that the clouds are often grey bodies and their emissivity is sensitive to the CDNC (refs. ^5,7,37^). The longwave indirect forcing at the MOSAiC location due to the blowing-snow-produced SSA is estimated by combining the measured cloud LWP with CDNC calculated offline using the GEOS-Chem-TOMAS model output (Methods)^38^. The emissivity is calculated twice, based on the CDNC calculated with and without the blowing-snow-produced SSA included, following the approach described in refs. ^5,39^ (Methods). The increase in downwelling cloud longwave radiation, derived from the difference between the two cloud emissivities, varies from 1.11 to 6.19 W m^−2^ (monthly mean values; Extended Data Table 2) from November to April. This increase of longwave radiation reflects the change in cloud emissivity due to increased CDNC under the same measured LWP (that is, first longwave aerosol indirect effect). Assuming the frequency distributions of LWP observed at the MOSAiC location are representative of the Arctic, we extend the first longwave indirect forcing calculation to the Arctic region. When the blowing-snow-produced SSA is included, the simulated CDNC increases by 10–35 cm^−3^ and the cloud droplet effective radius (*r*_e_) decreases by 0.45–0.90 μm (monthly mean values; Extended Data Fig. 4 and Extended Data Table 2) north of 70° N. These changes lead to an estimated downwelling longwave radiation increase of about +2.30 W m^−2^ under cloudy skies from November to April (Fig. 4b) north of 70° N. Higher CCN concentrations lead to more numerous and smaller cloud droplets, which are also expected to inhibit the formation of drizzle/rain and ice precipitation. A reduction in precipitation can result in higher LWP and longer cloud lifetime, further increasing the downwelling cloud longwave radiation. This second longwave indirect effect is probably substantial but is difficult to quantify and hence not investigated here. In summary, fine SSA produced by sublimation of saline blowing snow represents an important source of CCN in the Arctic during winter and spring. Our calculations suggest that the SSA can have a strong warming effect on the Arctic surface temperature by increasing cloud emissivity and probably the LWP and lifetime of clouds as well. The SSA production from blowing snow is also expected to play an important role in aerosol–cloud–climate interactions in the Antarctic, given the prevalence of sea ice and strong wind conditions.

## Methods

### Measurements

#### Measurement sites

Comprehensive measurements of meteorological conditions, aerosol, snowdrift density, cloud properties and open lead fraction were carried out in the framework of the MOSAiC expedition, which was designed to study Arctic climate change from multiple perspectives, including atmosphere, sea ice, snow, ocean, ecosystem and biogeochemistry^40^. The MOSAiC expedition took place in the central Arctic over a one-year period from September 2019 to October 2020. The track of the Polarstern icebreaker^41^, which served as the centre of operations during the MOSAiC expedition, and atmospheric measurement set-ups are summarized in ref. ^42^. Measurements employed in this study are briefly described below.

#### Meteorology parameters

Ambient temperature, wind speed and relative humidity with respect to liquid water (RH_w_) were measured at nominal heights of 2, 6 and 10 m from a tower installed at Met City, which was an installation on the sea ice 300–600 m from Polarstern^43^. The wind speed measurements were made by a three-dimensional sonic anemometer (Metek uSonic3 Cage MP), while the temperature and RH were measured by Vaisala HMT330/PTU300 sensors. The measured RH_w_ was then converted to relative humidity with respect to ice (RH_ice_)^44^.

#### Snowdrift density

The size distribution of airborne snow particles ranging from 36 to 490 µm was measured by an open-path Snow Particle Counter (SPC-95; Niigata Electric Co., refs. ^21,45^) and used to compute snowdrift density. During the MOSAiC expedition, two SPCs were set up at nominal heights of 0.1 and 10 m above the snow surface on the Met City tower. In this study, the snowdrift density (snow mass per volume of air) at 0.1 m is used to identify blowing-snow events except for November 2019, for which snowdrift density at 10 m is used instead due to the missing SPC data at 0.1 m.

#### Aerosol measurement

Aerosol properties were measured by the Aerosol Observing System of the US Department of Energy’s Atmospheric Radiation Measurement climate research facility^46^, which was positioned on the bow of Polarstern at 18 m above the sea ice surface. Aerosol samples were dried below 20% RH before being introduced to various instruments. The total number concentration of particles with diameters from 10 nm to 10 μm was measured by a Condensation Particle Counter (model 3772, TSI Inc.). Particle number size distribution was measured by a Scanning Mobility Particle Spectrometer (SMPS, model 3938; TSI Inc.) and an Ultra-High-Sensitivity Aerosol Spectrometer (UHSAS). Assuming spherical particles, we combine the SMPS and UHSAS measurements to generate particle size distribution with volume equivalent diameter (*d*_ve_) ranging from 10 to 1,000 nm. CCN concentration was measured by a Cloud Condensation Nuclei Counter^47^ consisting of two droplet activation columns. The supersaturation in the first column cycled through 0.12%, 0.27%, 0.54% and 0.76%, while the supersaturation in the second column was maintained at 0.49%. BC mass concentration was measured by a Single-Particle Soot Photometer^48^. The mass concentrations of non-refractory submicron aerosol species, including organics, sulfate, nitrate and ammonium, were measured by an ACSM (ref. ^49^). Aerosol hygroscopic growth at 85% RH was measured by a Humidified Tandem Differential Mobility Analyser (HTDMA, ref. ^50^). The measurement cycled through dry particle diameters of 50, 100, 150, 200 and 250 nm, and the measurement at each dry size took about 16 minutes. Super-micron particle number concentration (*N*_>1,000nm_) was measured using an Aerodynamic Particle Sizer (APS model 3321; TSI Inc.), which was operated in the Swiss measurement container^51^ adjacent to the Aerosol Observing System.

#### Cloud microphysical properties

Clouds were observed by a suite of instruments onboard Polarstern, which supported the derivation of a cloud phase and microphysical properties product^52,53^. Vertical profiles of cloud phase type are derived from radar, lidar, ceilometer, microwave radiometer and radiosonde measurements^54^, providing information for this study on the occurrence and vertical location of liquid water clouds. The total LWP within this framework was derived from a combination of microwave radiometer measurements^55–57^.

#### Arctic open leads

Daily Arctic sea ice leads are retrieved based on Moderate Resolution Imaging Spectroradiometer thermal infrared data^58^. Here sea ice leads are identified as substantial local surface temperature anomalies. A variety of lead metrics is used to distinguish between true leads and detection artefacts with the use of fuzzy logic. The resulting data yield daily sea ice lead maps at a resolution of 1 km^2^ from November to April.

### Identification of blowing-snow events (criteria)

Blowing-snow events are identified as periods when both the following criteria are met: (1) snowdrift density at 0.1 m above the snow surface is above 10^−5^ kg m^−3^ and (2) wind speed at 10 m above the snow surface exceeds the critical value, which was calculated from air temperature based on an empirical model^29^. Events shorter than 4 hours in duration (that is, brief spikes in snowdrift density) are excluded from further analysis. Adjacent events with brief gaps shorter than 2 hours are treated as a single continuous blowing-snow event. For November of 2019, the snowdrift density data at 0.1 m are not available, the blowing-snow events are instead defined as the periods when wind speed exceeds the threshold and fine-mode particle number concentration (*N*_10–300nm_) shows a strong enhancement (that is, a factor of 2) above the background, which is defined as 12-hour mean value of *N*_10–__300nm_ before wind speed exceeds the threshold. In addition, the snowdrift density measured at 10 m must exceed 10^−5^ kg m^−3^ for at least 1 hour during the identified events to confirm the presence of blowing snow. On the basis of the above criteria, blowing-snow events occurred more than 20% of the time from November 2019 to April 2020 (Extended Data Table 1). The wind speed threshold for the onset of blowing snow (an increase in snowdrift density) at the MOSAiC location generally matches, but is slightly lower than, the value proposed in ref. ^29^ (Ranjithkumar et al., manuscript in preparation). Moreover, the threshold wind speed for the onset of blowing snow is always higher than that for maintaining blowing snow because the additional wind stress is needed to overcome snow crystal bonding and initiate saltation^59^. Therefore, the stricter criteria applied here probably lead to an underestimate of blowing-snow event time in the Arctic.

During MOSAiC, aerosol measurements were occasionally influenced by local primary pollution, including ship emissions from Polarstern and human activities onboard and near the ship^60^. In addition, there are occasional gaps in the aerosol data, partially due to the challenges in conducting long-term measurements in the central Arctic. To statistically examine the aerosol properties during the blowing-snow events, we classify the period from November 2019 to April 2020 into four categories: local primary pollution periods (identified by visual detection and explained in ref. ^60^), periods with no aerosol data, blowing-snow events with valid aerosol data and non-blowing-snow periods with valid aerosol data. These four categories respectively account for about 22.13%, 19.14%, 13.07% and 45.6% of the six months (Extended Data Fig. 1). The shortest duration of a blowing-snow event is about 7 hours, and the longest event lasted for almost three days due to sustained strong wind. Both fine-mode and super-micron particle number concentrations and *N*_CCN_ are substantially higher during blowing-snow events (Extended Data Fig. 1). The increases of particle and CCN concentrations vary from event to event and can be up to more than ten times higher than those during non-blowing-snow periods.

### Derivation of particle hygroscopicity

The humidified particle size distribution measured by the HTDMA is first converted to a normalized growth factor (GF = diameter after humidification/initial dry diameter) distribution. We use up to three Gaussian distributions to fit each GF distribution. Poor fits with the sum of squared residuals greater than 20 are excluded from the analysis. About 95% of the time, only the hydrophilic mode(s) (that is, mode with GF greater than 1.15) is present, indicating internally mixed aerosol with respect to hygroscopicity. The growth factor is calculated by averaging the hydrophilic mode(s). The hygroscopicity parameter under sub-saturation (κ_GF_) is then calculated from the average growth factor^31^. The contour plot of particle growth factor distribution and the averaged growth factor are present in Supplementary Fig. 4. As shown in the main text, SSA dominates fine-mode aerosol during blowing-snow events. Because the size of non-spherical dry sea salt particles (shape factor of 1.05–1.10) is overestimated by the first DMA of the HTDMA, the calculated GF probably represents a lower limit, leading to an underestimation of the particle hygroscopicity during blowing-snow events. Given the difference in hygroscopicity between background aerosol and blowing-snow-produced SSA, it is somewhat counterintuitive that the GF distribution observed during the blowing-snow events mostly exhibits a single hydrophilic mode. This is because SSA dominates the aerosol population during blowing-snow events. As a result, the GF distribution of the background particles is completely overshadowed by the SSA, leading to a slightly broadened GF mode as shown in Supplementary Fig. 4.

Particle hygroscopicity under supersaturation is derived by combining particle number size distribution and *N*_CCN_^61,62^. As described above, aerosols are mostly internally mixed based on the HTDMA measurements (that is, particles of the same diameter have similar hygroscopicity). Therefore, for a given supersaturation (*s*), the critical particle activation diameter (*d*_c_) can be derived using the following equation:

1 $${N}_{\mathrm{CCN}}\left(s\right)={\int }_{{d}_\mathrm{c}\left(s\right)}^{+{\rm{\infty }}}n\left({D}_\mathrm{p}\right)\mathrm{d}{D}_\mathrm{p}$$

where *N*_CCN_ and *n*(*D*_p_) represent measured CCN concentration and particle number size distribution, respectively. Particle hygroscopicity κ_CCN_(*s*) is then derived from *d*_c_(*s*) and *s* based on κ-Köhler theory^31^. The uncertainty in derived κ_CCN_(*s*) originates from the uncertainties in the size distributions measured by SMPS and UHSAS, supersaturation inside the CCN counter and measured *N*_CCN_. The uncertainty of κ_CCN_(*s*) is quantified using a Monte Carlo approach^62^, and the uncertainty of κ_CCN,0.75%_ is shown using error bars as an example in Supplementary Fig. 1. As the *d*_ve_ of non-spherical dry sea salt particles is overestimated by the SMPS, the above method probably underestimates κ_CCN_(*s*) values during blowing-snow events, when SSA dominates the aerosol population.

### Global model simulation

The GEOS-Chem chemical transport model (version 13.2.1, http://wiki.seas.harvard.edu/geos-chem/index.php/GEOS-Chem_13.2.1; https://zenodo.org/record/5500717#.YpjnyC-cbxg) was used to simulate the blowing-snow-produced SSA and central Arctic aerosols. The model was coupled to the TwO-Moment Aerosol Sectional (TOMAS) microphysics scheme^38,63,64^ to represent aerosol particles with diameters ranging from 3 nm to about 10 μm using a set of 15-size bins. All simulations were conducted with a 4° (latitude) × 5° (longitude) resolution due to the computational expense of TOMAS. The simulation used 47 vertical levels from the Earth’s surface to 0.01 hPa. Meteorological fields from Modern-Era Retrospective Analysis for Research and Applications, Version 2 (MERRA-2)^65^ were used to drive the simulations. MERRA-2 has a spatial resolution of 1/2° (latitude) by 2/3° (longitude) with 72 vertical levels extending to 0.01 hPa and was re-gridded to the GEOS-Chem-TOMAS resolution of our simulations. For wind speed and temperature, two key parameters of blowing-snow parameterization, good agreements are found between the measurements and the MERRA-2 data (Extended Data Fig. 2). RH_ice_ from the reanalysis data generally agrees with the measurement (Extended Data Fig. 2).

The GEOS-Chem-TOMAS model includes parameterizations for the processes of particle nucleation, coagulation, vapour condensation, wet removal^66,67^ and dry deposition^68^. These parameterizations allow for the simulation of size-resolved sulfate, organics, BC, sea salt, dust and aerosol water within the full tropospheric aerosol chemistry scheme of GEOS-Chem. Removals of gas-phase species are represented as in ref. ^69^. We used the Community Emissions Data System for global anthropogenic sources of NO_*x*_, CO, SO_2_, NH_3_, non-methane VOCs, BC and organic carbon^70^. The Global Fire Emissions Database (GFED4) was used for biomass-burning emissions^71^ and dust emissions following ref. ^72^.

Sea salt emissions from the open ocean are represented using the parameterization developed by ref. ^73^. This scheme includes a temperature-dependent modification of the sea salt emissions parameterization^74^. We also implemented the parameterization of size-resolved blowing-snow SSA emissions, which was previously developed by ref. ^17^ based on the work of ref. ^20^. The size-resolved emission is distributed across the TOMAS size bins. The median snow salinity observed at the MOSAiC location from November to April is 0.10 practical salinity units (PSU) (522 samples). A snow salinity of 0.10 PSU over first-year Arctic sea ice and 0.05 PSU over multi-year Arctic sea ice were therefore used in our simulations, the same as the previous study^17^. We assumed that snow particle size distribution follows a modified gamma function^20^. The two parameters (α and β) of the gamma function were parameterized as functions of wind speed based on measurements during MOSAiC (Ranjithkumar et al., manuscript in preparation) and were implemented in the model. As there were no direct measurements of the number of sea salt particles produced by each sublimated snow particle (that is, NP), we carried out simulations using both NP = 1 and NP = 5, as suggested in refs. ^17,27^, then compared the simulation results with aerosol measurements to constrain the NP value. Because the simulation with NP = 5 shows much better agreement with the measurements than the simulation with NP = 1, the NP = 5 simulation is designated as the base simulation in this study (Supplementary Discussion 4). We note that a previous modelling study^17^ also found that NP = 5 is a more appropriate value than NP = 1. The sensitivity of simulated particle concentrations to the salinities was examined, and we found that reducing the salinities by half only slightly changes the simulated submicron particle number size distribution and CCN concentration (Supplementary Discussion 5). The comparison between the aerosol concentrations from the base simulation and the measurements during MOSAiC is detailed below.

Extended Data Fig. 2 shows the comparison between the measured total particle number concentration (*N*_total_) and GEOS-Chem-TOMAS-simulated *N*_total_ (base simulation). The inclusion of blowing-snow-produced SSA in the simulation better captures the episodic enhancement of particle number concentrations during blowing-snow events. After blowing-snow-produced SSA is included in the simulation, the correlation coefficient (*R*) between the simulated and measured *N*_total_ (4 h mean values) increases from 0.43 (*p* value = 1.33 × 10^−35^) to 0.53 (*p* value = 1.09 × 10^−56^) for the period between November and April. During some of the blowing-snow events, the simulated increase of particle concentration is more gradual compared with the observation (Extended Data Fig. 2). Potential causes of the differences may include the relatively coarse model spatial resolution, the uncertainties associated with the blowing-snow parameterization^27^ and the uncertainties in meteorological conditions. For example, the emissions flux of blowing-snow-produced SSA is a superlinear function of wind speed. Coarse model resolution smooths the wind speed variability and probably leads to underprediction of the emissions flux. The simulation that includes blowing-snow-produced SSA also better reproduces the observed particle number size distribution, especially in the Aitken mode particle size range. The simulation also appears to overestimate the accumulation mode particle concentration even when blowing snow is not included (Extended Data Fig. 3), possibly due to excessive cloud processing and insufficient wet scavenging in the model^38,75^. Future work will include further development of the blowing-snow SSA parameterization and simulations with increased model spatial resolutions.

### Estimation of the impact on cloud emissivity and surface warming

The effect of blowing-snow-produced SSA on longwave emission of Arctic clouds is evaluated from the base simulation using the same approach described in refs. ^5,39^, which reported the change of cloud longwave radiation due to Arctic haze. We first estimate the cloud longwave radiative effect at the MOSAiC location by combining the measured time series of LWP and model-simulated CDNC. The time series of CDNC was simulated twice, with and without blowing-snow-produced SSA included. The cloud droplet effective radius (*r*_e_) is assumed as 10 μm (refs. ^76,77^) when blowing-snow-produced SSA is included. We then estimate the corresponding *r*_e_ when blowing-snow-produced particles are excluded based on the change in CDNC, assuming the same measured LWP and thus liquid water content. The broadband cloud longwave emissivities (ε) with and without blowing-snow-produced SSA included are calculated using *r*_e_, measured LWP and cloud temperature^5,39^. The increases in downwelling cloud longwave radiation, derived from the difference between the two cloud emissivities, ranges from 1.11 to 6.19 W m^−2^ under cloudy skies from November to April (monthly mean values; Extended Data Table 2). We note this increase of longwave radiation reflects the change in cloud emissivity due to increased CDNC only (that is, first longwave aerosol indirect effect; LWP is assumed not to be affected by the change in CDNC). A cloudy sky condition at the MOSAiC location is identified when a minimum of two data points in the vertical column below 3 km indicates the presence of liquid, drizzle, liquid cloud and drizzle, rain or mixed-phase clouds using the cloud phase classification^54^. The monthly mean percentages of cloudy sky conditions are about 53%, 41%, 33%, 26%, 26% and 44% for the respective months from November 2019 to April 2020 (Extended Data Table 2).

Assuming the frequency distributions of LWP observed at the MOSAiC location are representative of the Arctic region, we extend the first longwave indirect effect estimation to the Arctic region. For each month from November 2019 to April 2020, the frequency distribution of LWP under cloud–sky conditions is first derived from the measurement at the MOSAiC location. For each grid box, the change in *r*_e_ is estimated using the monthly average CDNC with and without blowing-snow-produced SSA included. The monthly mean first longwave aerosol indirect effect for the grid box is then derived from the increase in cloud emissivity calculated from the change in *r*_e_ and different LWP values over the range observed, weighted by the monthly frequency distribution of LWP. The monthly LWP frequency distributions under cloudy skies are shown in Extended Data Fig. 4.

## Online content

Any methods, additional references, Nature Portfolio reporting summaries, source data, extended data, supplementary information, acknowledgements, peer review information; details of author contributions and competing interests; and statements of data and code availability are available at 10.1038/s41561-023-01254-8.

### Supplementary information

Supplementary Information Supplementary Discussions 1–5, Table 1, Figs. 1–8 and References.

## Figures and Tables

**Extended Data Table 1:**
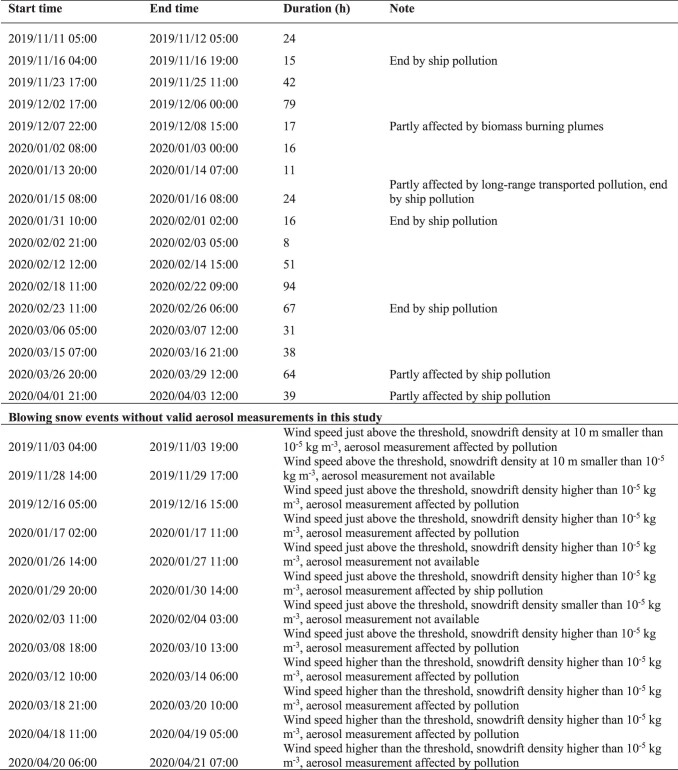
Summary of blowing snow events

Blowing snow events with valid aerosol measurements are summarized in the upper part of the table. Occasional and/or partial influence of aerosol measurements by long-range transported or local primary pollution is noted in the last column. Blowing snow events influenced by local primary pollution during the entire period and events with missing AOS aerosol measurements (including particle number size distribution, particle number concentration, and CCN concentration) are summarized in the lower part of the table. These events are not included in the statistical analysis of aerosol properties.

**Extended Data Table 2:**
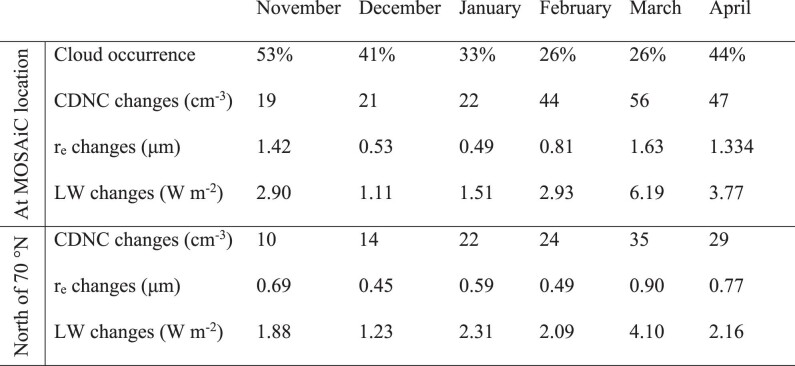
Cloud occurrence and changes in cloud microphysical and longwave radiative properties attributed to the blowing-snow-produced sea salt aerosols

The monthly mean values of cloud occurrence at the MOSAiC location are based on remote sensing measurements. The changes in cloud droplet number concentration (CDNC) and cloud effective radius (*r*_e_) are based on model simulations. The longwave (LW) downwelling radiation flux at the MOSAiC location and in the Arctic region (north of 80 °N and 60 °N) attributed to the blowing-snow-produced SSA is derived from the changes in *r*_e_ and measured liquid water path (LWP).

## Data Availability

Total particle number concentration, particle number size distribution, cloud condensation nuclei number concentration, particle hygroscopicity, black carbon and chemical composition data are available in DOE ARM Data Archive (https://adc.arm.gov/discovery/#/results/s::MOSAiC). Cloud properties data are available in DOE ARM Data Archive and PANGAEA (10.5439/1871015; 10.1594/PANGAEA.941389). Met City meteorological data are available from the National Science Foundation’s Arctic Data Center (10.18739/A2VM42Z5F). Snowdrift data are available from the UK Polar Data Center (10.5285/7d8e401b-2c75-4ee4-a753-c24b7e91e6e9). The Arctic open lead fraction datasets are available in PANGAEA (10.1594/PANGAEA.955561). The APS data are available in PANGAEA (https://doi.pangaea.de/10.1594/PANGAEA.960923).

## Code availability

The GEOS-Chem-TOMAS model version used in this study is available at https://zenodo.org/record/5500717#.YpjnyC-cbxg. The MATLAB codes used to analyse the data are available upon request.
