# Supplementary material for: Arctic warming by abundant fine sea salt aerosols from blowing snow
Source: Nat Geosci. 2023 Sep 4;16(9):768–74. doi: 10.1038/s41561-023-01254-8 (PMC10482690; doi:10.1038/s41561-023-01254-8)
Supplement: Supplementary file 1 — Supplementary Discussions 1–5, Table 1, Figs. 1–8 and References. [file 41561_2023_1254_MOESM1_ESM.pdf]

nature geoscience

Article

https://doi.org/10.1038/s41561-023-01254-8

Arctic warming by abundant fine sea salt aerosols from blowing snow

In the format provided by the authors and unedited

Supplementary Materials

The Supplementary file includes:

Supplementary Discussion 1-5;

Supplementary Table 1;

Supplementary Figures 1-8;

Supplementary References.

Supplementary Discussion

1: Estimation of the blowing snow particle number size distribution for event 3

Given their relatively low concentrations, background aerosols are expected to have a minor contribution to the aerosol population during the blowing snow events 1 and 2. However, during the 3rd blowing snow event from 22:00 on 7 December to 15:00 on 8 December, the influence of long-range transported biomass burning plumes[30] is substantial, as evidenced by the elevated BC mass concentration (Fig. 2c). To evaluate the concentration of blowing-snow-produced aerosols during event 3, we subtract the contribution of long-range transported biomass burning aerosol from the total aerosol population using the following approach. On 11 December, the BC mass concentration is about 40-70 ng cm^{-3}, nearly the highest level during this biomass burning event. In addition, derived sea salt mass concentration is negligible, indicating that the aerosol observed on 11 December is dominated by biomass burning pollution. The derivation of the sea salt mass concentration is detailed in the section "Consistencies among aerosol measurements and the derivation of sea salt mass concentration" in Supplementary Discussion 2. The average particle number size distribution and CCN concentration on 11 December are therefore considered as the elevated "background" due to the influence of the biomass burning aerosol. The population of blowing-snow-produced aerosol is then derived by subtracting the elevated background from the total aerosol measured during the blowing snow event 3 on 8 December, when BC mass concentration is above 40 ng cm^{-3}.

2: Consistencies among aerosol measurements and the derivation of sea salt mass concentration

We examine the consistencies among measured particle size distribution, mass concentrations and particle hygroscopicity. These consistency checks (i.e., closure studies) provide additional high-

level data quality assurance. Consistency between particle size distribution and mass concentrations is examined. The particle volume size distribution is first derived from the measured particle number size distribution. ACSM measures non-refractory components of aerosol particles with vacuum aerodynamic diameter (d_{va}) up to 1000 nm. The major aerosol species during MOSAiC are expected to include sulfate, organics and sea salt. The densities of sea salt, ammonium sulfate, glucose and sodium alginate (the latter two are major marine organic aerosols[78,79]) are 2.16, 1.77, 1.56 and 1.00 g cm^{-3}, respectively. The shape factors of sea salt[80], ammonium sulfate[81] and organics[81] are about 1.05-1.10, 1.03-1.07 and 1, respectively. For spherical particles (shape factor = 1) with a density of 1.6 g cm^{-3}, the volume equivalent particle diameter (d_{ve}) corresponding to the ACSM upper size limit is calculated as 625 nm. The total submicron (i.e., d_{va}<1000 nm) aerosol mass concentration is then derived by integrating particle volume size distribution from 10 to 625 nm ($M_{10\text{-}625nm}$, Fig. 2c). The impact of assumed particle density and shape factor on derived mass concentration is discussed below. During non-blowing snow periods, $M_{10\text{-}625nm}$ shows a strong correlation (R^2=0.83, p-value=5.29×10^{-53}, Supplementary Fig. 1a) and agrees well with the non-refractory submicron mass concentration measured by ACSM (M_{ACSM}, sum of mass concentrations of sulfate, organics, ammonium and nitrate). During the blowing snow events, $M_{10\text{-}625nm}$ is substantially higher than M_{ACSM} due to the presence of SSA. As sea salt is refractory and cannot be reliably quantified by ACSM, sea salt mass concentration is therefore derived as the difference between $M_{10\text{-}625nm}$ and M_{ACSM}. The average sea salt mass concentrations during the blowing-snow events and non-blowing periods are shown as magenta bars in Fig. 2e. We note that the sea salt mass concentration derived using the above approach could potentially include contributions from refractory primary marine organics.

The impact of assumed particle density and shape factor on derived mass concentration are examined separately. We found the derived submicron mass concentration is insensitive to assumed particle density because the increase of derived mass concentration due to higher particle density is largely offset by the reduction of the upper limit of d_{ve} over the expected density range of 1.5 – 2 g cm^{-3}. For example, the upper limit of d_{ve} decreases from 625 nm to 500 nm when particle density increases from 1.6 to 2 g cm^{-3}. As a result, using a density of 2 instead of 1.6 g cm^{-3} leads to an average of ~ 7.1% increase in derived mass concentration. The impact of particle shape on derived mass concentration is also very minor. For particles with a density of 1.6 g cm^{-3}, using a shape factor of 1.05 instead of 1.00 leads to an average of ~ 6.8% decrease in the derived mass concentration.

To check the consistency between hygroscopicity and composition measurements, we derive the aerosol hygroscopicity from the bulk submicron composition using the mass concentrations of sulfate, organics, ammonium, nitrate and sea salt. We employ a simplified ion-pairing scheme with a direct analytical solution to calculate the number of moles of $(NH_4)_2SO_4$, NH_4HSO_4, H_2SO_4, NH_4NO_3, organics and NaCl[82]. As the aerosol is mostly internally mixed, κ_{CCN} and κ_{GF} are derived as the volume average of κ values for the participating species[31,32,82] (hollow squares in Supplementary Fig. 1c,d). We note that particles larger than ~250 nm dominated the bulk submicron aerosol composition. Nevertheless, κ_{GF} derived from the bulk composition shows a good agreement (R^2=0.65, p-value=9.84×10^{-45}) with the measured $\kappa_{GF,250nm}$ (Supplementary Fig. 1b). The agreements between derived and measured mass concentrations and aerosol hygroscopicities indicate consistencies among the different measurements and high quality of the datasets.

3: Contribution of the sea salt aerosols emitted from open leads

Arctic open leads are considered an important local source of SSA based on measurements in the Alaskan Arctic during winter and above the Arctic Ocean up to 88 °N in summer[18,83]. The contribution of SSA generated from open leads during the blowing snow events is investigated. Four-day back-trajectories of air masses arriving at the MOSAiC location are simulated using the HYSPLIT (HYbrid Single-Particle Lagrangian Integrated Trajectory) model[84]. The back-trajectories are originated at 100 m above sea ice every hour at the MOSAiC location. The fraction of the time that the air mass is exposed to open leads during the 4 days, referred to as the "average open lead fraction" is then calculated by combining each back-trajectory with the open lead fraction map. The air mass is considered "exposed to open leads" when the air mass is above open leads and the altitude of the air mass is below 300 m (roughly the boundary layer height during winter and spring in the Arctic). Trajectories with total precipitation during the 4-day period above 20 mm (i.e., strong wet removal) are excluded from further analysis. Daily mean values are then derived by averaging the average open lead fractions of 24 hourly back-trajectories for each day (orange bars in Supplementary Fig. 2a,b). The elevation of snowdrift density (i.e., presence of blowing snow) coincides with high wind speed exceeding the critical threshold (Supplementary Fig. 2a,b) and fine-mode particle number concentration ($N_{10\text{-}300\mathrm{nm}}$) is strongly enhanced during the blowing snow events (Extended Data Fig. 1). In contrast, the variation of the daily mean open lead fraction shows no clear correlation with wind speed, suggesting that the open lead fraction does not explain the episodic nature of the elevated fine-mode particle concentration.

We then analyze the particle emission from open leads along the airmass trajectories. Particle net emission flux from open leads is parameterized as a function of wind speed at 10 m height[83]. Here the horizontal wind speed from the back-trajectories is used for the parameterization. The particle emission flux from open leads along an airmass trajectory, referred to as "weighted open lead flux",

is derived by averaging the emission flux along the trajectory, weighted by the local open lead fraction. We classify the weighted open lead flux into 3 levels (0-0.004, 0.004-0.008 and >0.08×10^6 m^{-2} s^{-1}). The $N_{10\text{-}300nm}$ frequency distributions under three levels of weighted open lead flux are very similar. In addition, no correlation between $N_{10\text{-}300nm}$ and the weighted open lead flux is found (Supplementary Fig. 3).

There are uncertainties associated with the weighted open lead flux derived above. The sea-ice leads are identified as significant positive local surface temperature anomalies. Some of the leads may be covered by thin ice[85], therefore, the open lead fraction and thus the particle flux could be overestimated. Uncertainty in the wind fields such as sub-grid variability could lead to uncertainty in calculated back-trajectories. The originating altitude of the back-trajectory could also lead to additional uncertainty. We repeat the above analysis using the 1-, 2-, 4- and 6-day back-trajectories originated at altitudes of both 100 and 200 m, and find no correlations between $N_{10\text{-}300nm}$ and the weighted open lead fluxes for different originating altitudes or over different lengths of back-trajectory.

Based on current knowledge, the production of SSA from open leads follows similar mechanisms as sea spray aerosol generation from the open ocean (i.e., wave-breaking processes resulting in bubble bursting at the ocean surface, creating film and jet drops that form sea spray aerosol[24]). Previous studies show that under the same wind speed, the particle emission flux over the open leads is lower than that over the open ocean[83], due to the reduced wind fetch over open leads. Measurements over open oceans typically show a much lower fine mode SSA number concentration under similar wind conditions as during the blowing snow events observed in this study[23,25]. The spatial coverage of open leads is much lower than sea ice in the central Arctic during winter/spring. While particles generated from open leads likely contribute to the aerosol population

during the events, the lack of correlation between $N_{10\text{-}300\text{nm}}$ and the weighted open lead flux indicates that open leads are unlikely the major source of fine mode particles. The coincidence of enhanced $N_{10\text{-}300\text{nm}}$ with the presence of blowing snow suggests that the sublimation of blowing snow is likely the dominant source of fine-mode particles during the events.

4: Constraining the value of NP using MOSAiC measurements

We carried out simulations with NP=5 and NP=1 and compared them with aerosol measurements during MOSAiC to constrain the NP value. The simulation with NP=5 shows much better agreement with measured total particle number concentrations (Supplementary Fig. 5) and the particle number size distributions (Extended Data Fig. 3 and Supplementary Fig. 6) than the simulation with NP=1. The mean fractional bias (MFB) of simulated total particle number concentration during blowing snow events is 9.7% and −27.4% for NP=5 and NP=1, respectively. The very negative MFB of NP=1 simulation indicates a severe underestimation of blowing-snow-produced sea salt particle concentration during the blowing snow events. This is also consistent with the underestimate of particle size distribution in the Aitken mode size range during most months (Supplementary Fig. 6). In comparison, NP=5 simulation reasonably reproduces the Aitken mode particle concentration during all months except March, when the Aitken mode concentration is overestimated. Based on the above analyses, we designated NP=5 simulation as the base simulation in this study and used NP=5 in additional simulations for the salinity sensitivity test described below.

5: Sensitivity test of snow salinity in blowing snow parameters

To examine the sensitivity of simulated particle concentrations to the salinities, we carried out simulation (i.e., low salinity simulation) using FYI and MYI salinities of 0.05 and 0.025 psu, respectively, a factor of 2 decreases from the base values. The 0.05 psu for the FYI is about the

lowest mean value when the snow depth is above 20 cm (ref. [86]). Lower salinities reduce the size of generated sea salt particles, thus decreasing their contribution to the CCN concentration and the longwave radiative effect. Therefore, this sensitivity test provides the lower limit estimate of the CCN production from the blowing snow. We found that reducing the salinities by half only slightly changes the simulated submicron particle number size distribution (Extended Data Fig. 3 and Supplementary Fig. 7). The lower salinities lead to a slight decrease (4.9%) of simulated CCN concentration at a supersaturation of 0.30% (Supplementary Fig. 8), consistent with the minor impact on simulated size distribution. The above results suggest that the salinities employed in the base simulation do not lead to a substantial overestimation of the CCN population and the longwave radiative effect.

Supplementary Table

Supplementary Tab. 1 | Summary of the sample size used to derive the boxplot in Extended Data Fig. 1

Start Time	End Time	Mean Time	Classification	n for $N_{CCN,0.27\%}$	n for $N_{10\text{-}300nm}$	n for $N_{>1000nm}$
2019/11/11 00:00	2019/11/11 05:00	2019/11/11 02:30	Non Blowing snow	56	574	3654
2019/11/11 05:00	2019/11/12 05:00	2019/11/11 17:00	Blowing snow	36	283	1430
2019/11/12 05:00	2019/11/16 04:00	2019/11/14 04:30	Non Blowing snow	126	1054	5397
2019/11/16 04:00	2019/11/16 19:00	2019/11/16 11:30	Blowing snow	22	181	901
2019/11/16 19:00	2019/11/23 17:00	2019/11/20 06:00	Non Blowing snow	56	466	2447
2019/11/23 17:00	2019/11/25 11:00	2019/11/24 14:00	Blowing snow	61	495	2488
2019/11/25 11:00	2019/12/02 17:00	2019/11/29 02:00	Non Blowing snow	75	587	3029
2019/12/02 17:00	2019/12/06 00:00	2019/12/04 08:30	Blowing snow	117	933	4667
2019/12/06 00:00	2019/12/07 22:00	2019/12/06 23:00	Non Blowing snow	52	431	2235
2019/12/07 22:00	2019/12/08 15:00	2019/12/08 06:30	Blowing snow	25	200	1021
2019/12/08 15:00	2020/01/02 08:00	2019/12/20 23:30	Non Blowing snow	539	4286	22775
2020/01/02 08:00	2020/01/03 00:00	2020/01/02 16:00	Blowing snow	23	91	947
2020/01/03 00:00	2020/01/13 20:00	2020/01/08 10:00	Non Blowing snow	266	2017	11151
2020/01/13 20:00	2020/01/14 07:00	2020/01/14 01:30	Blowing snow	15	108	652
2020/01/14 07:00	2020/01/15 08:00	2020/01/14 19:30	Non Blowing snow	36	293	1490
2020/01/15 08:00	2020/01/16 08:00	2020/01/15 20:00	Blowing snow	30	254	1354
2020/01/16 08:00	2020/01/31 10:00	2020/01/23 21:00	Non Blowing snow	270	2168	11270
2020/01/31 10:00	2020/02/01 02:00	2020/01/31 18:00	Blowing snow	23	188	954
2020/02/01 02:00	2020/02/02 21:00	2020/02/01 23:30	Non Blowing snow	12	96	581
2020/02/02 21:00	2020/02/03 05:00	2020/02/03 01:00	Blowing snow	12	94	479
2020/02/03 05:00	2020/02/12 12:00	2020/02/07 20:30	Non Blowing snow	325	2425	13262
2020/02/12 12:00	2020/02/14 15:00	2020/02/13 13:30	Blowing snow	75	602	3041
2020/02/14 15:00	2020/02/18 11:00	2020/02/16 13:00	Non Blowing snow	127	999	5146
2020/02/18 11:00	2020/02/22 09:00	2020/02/20 10:00	Blowing snow	125	1001	5129
2020/02/22 09:00	2020/02/23 11:00	2020/02/22 22:00	Non Blowing snow	39	308	1553
2020/02/23 11:00	2020/02/26 06:00	2020/02/24 20:30	Blowing snow	93	751	3945
2020/02/26 06:00	2020/03/06 05:00	2020/03/01 17:30	Non Blowing snow	161	1225	7045
2020/03/06 05:00	2020/03/07 12:00	2020/03/06 20:30	Blowing snow	41	279	1682
2020/03/07 12:00	2020/03/15 07:00	2020/03/11 09:30	Non Blowing snow	87	623	3594
2020/03/15 07:00	2020/03/16 21:00	2020/03/16 02:00	Blowing snow	54	368	2254
2020/03/16 21:00	2020/03/26 20:00	2020/03/21 20:30	Non Blowing snow	148	1165	6153
2020/03/26 20:00	2020/03/29 12:00	2020/03/28 04:00	Blowing snow	21	151	1201
2020/03/29 12:00	2020/04/01 21:00	2020/03/31 04:30	Non Blowing snow	29	238	1225
2020/04/01 21:00	2020/04/03 12:00	2020/04/02 16:30	Blowing snow	52	414	2130
2020/04/03 12:00	2020/05/01 00:00	2020/04/17 06:00	Non Blowing snow	413	3327	17659

Supplementary Figures

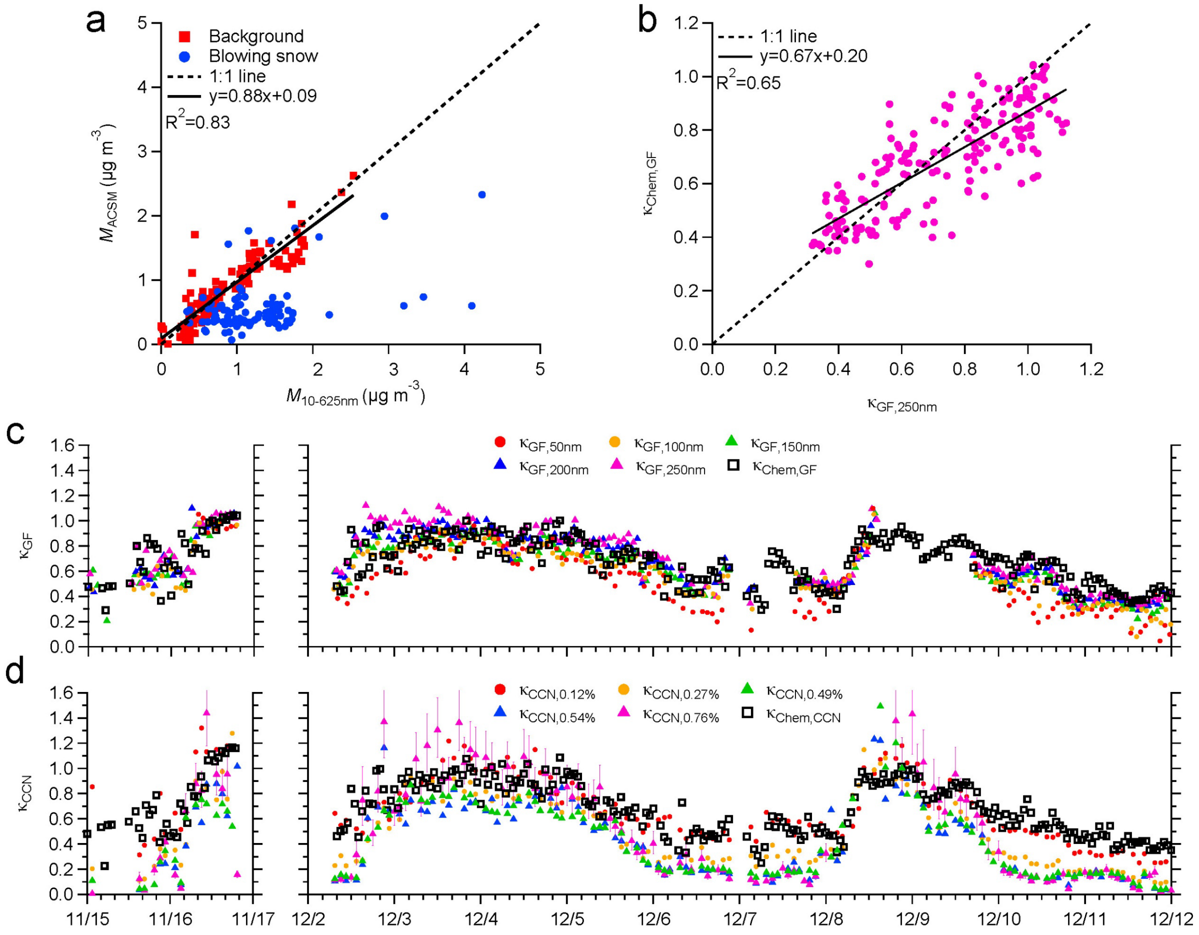

Supplementary Fig. 1 | Closure study between particle number size distribution, chemical composition and particle hygroscopicity. a, Comparison of submicron non-refractory mass concentration (i.e., the sum of sulfate, organics, ammonium and nitrate) measured by ACSM (M_{ACSM}) with mass concentration of particles with volume equivalent diameter between 10 to 625 nm derived from particle size distribution ($M_{\mathrm{10\text{-}625nm}}$) during the non-blowing snow periods (red squares) and blowing snow events (blue dots). During non-blowing snow periods, the square of the correlation coefficient (R^2) between M_{ACSM} and $M_{\mathrm{10\text{-}625nm}}$ is 0.83. **b**, Comparison of particle hygroscopicity derived from chemical composition under sub-saturation ($\kappa_{\mathrm{Chem,GF}}$) with the particle hygroscopicity derived from particle growth factor for 250 nm particles ($\kappa_{\mathrm{GF,250nm}}$). The

square of the R^2 between $\kappa_{Chem,GF}$ and $\kappa_{GF,250nm}$ is 0.65. **c**, Time series of aerosol hygroscopicity derived from growth factor (κ_{GF}) for particles of 50, 100, 150, 200 and 250 nm (κ_{GF}) and aerosol hygroscopicity under sub-saturation derived from bulk submicron aerosol composition ($\kappa_{Chem,GF}$). **d**, Time series of aerosol hygroscopicity under supersaturation of 0.12%, 0.27%, 0.49%, 0.54% and 0.76% (κ_{CCN}) derived from cloud condensation nuclei activation and aerosol hygroscopicity under supersaturation derived from bulk submicron aerosol composition ($\kappa_{Chem,CCN}$). Error bars represent the measurement uncertainty of $\kappa_{CCN,0.75\%}$ (explained in Methods).

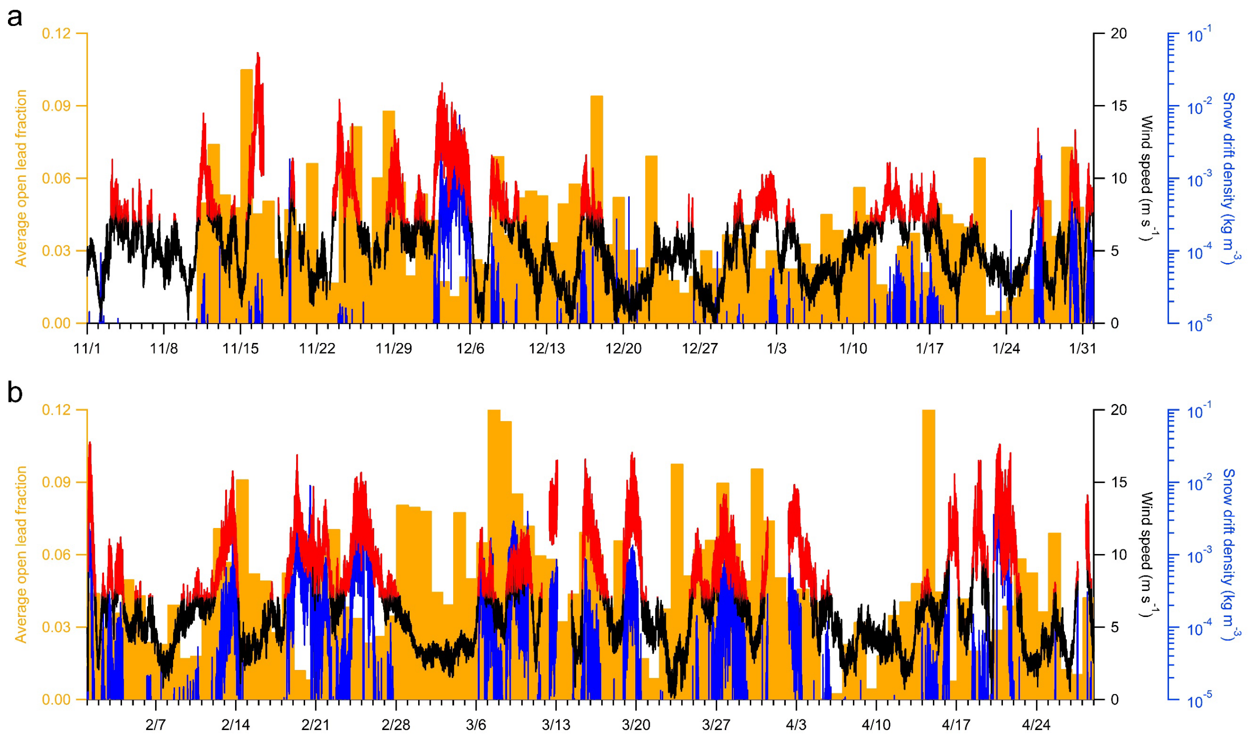

Supplementary Fig. 2 | Blowing snow events and the variation of Arctic open lead fraction. The time series (from November to January in panel **a** and February to April in panel **b**) of the daily mean value of the average open lead fraction along the backward trajectory (orange bars), snowdrift density (blue line), and wind speed above the critical threshold (red line) and below the threshold (black line).

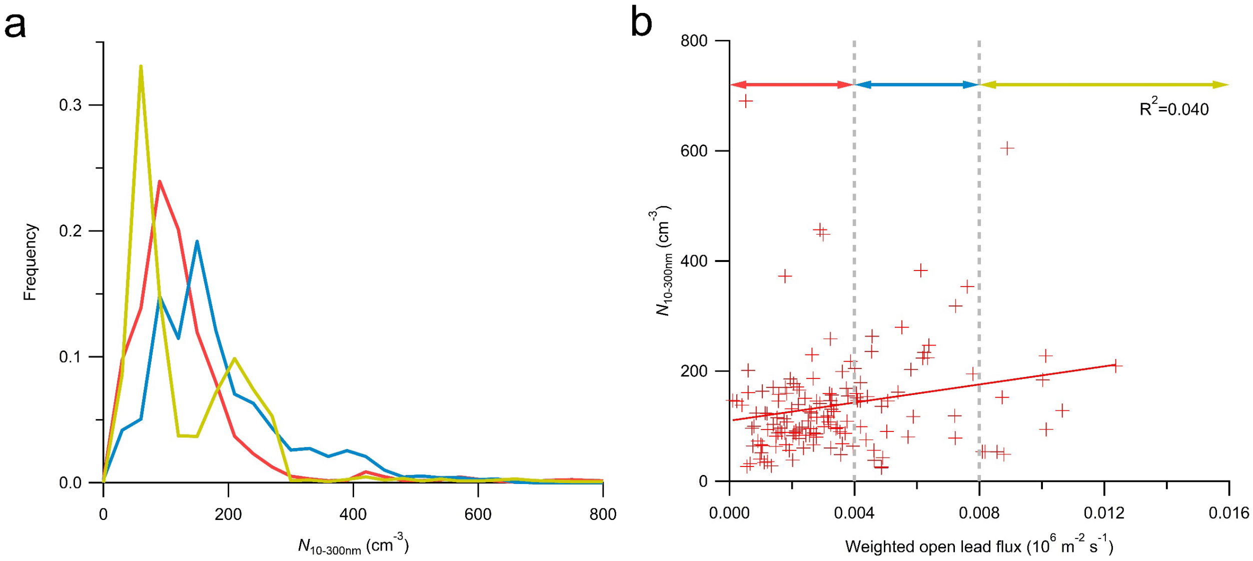

Supplementary Fig. 3 | The relationship between fine-mode particle number concentration and weighted particle emission flux from open leads along airmass back-trajectory. a, The frequency distribution of fine-mode particle number concentration ($N_{10\text{-}300nm}$) for three different ranges of weighted particle emission flux from open leads along airmass trajectory (weighted open lead flux, 0-0.004×10^6 m^{-2} s^{-1} in red, 0.004-0.008×10^6 m^{-2} s^{-1} in blue and >0.008×10^6 m^{-2} s^{-1} in yellow). **b**, The correlation between $N_{10\text{-}300nm}$ and the weighted open lead particle flux.

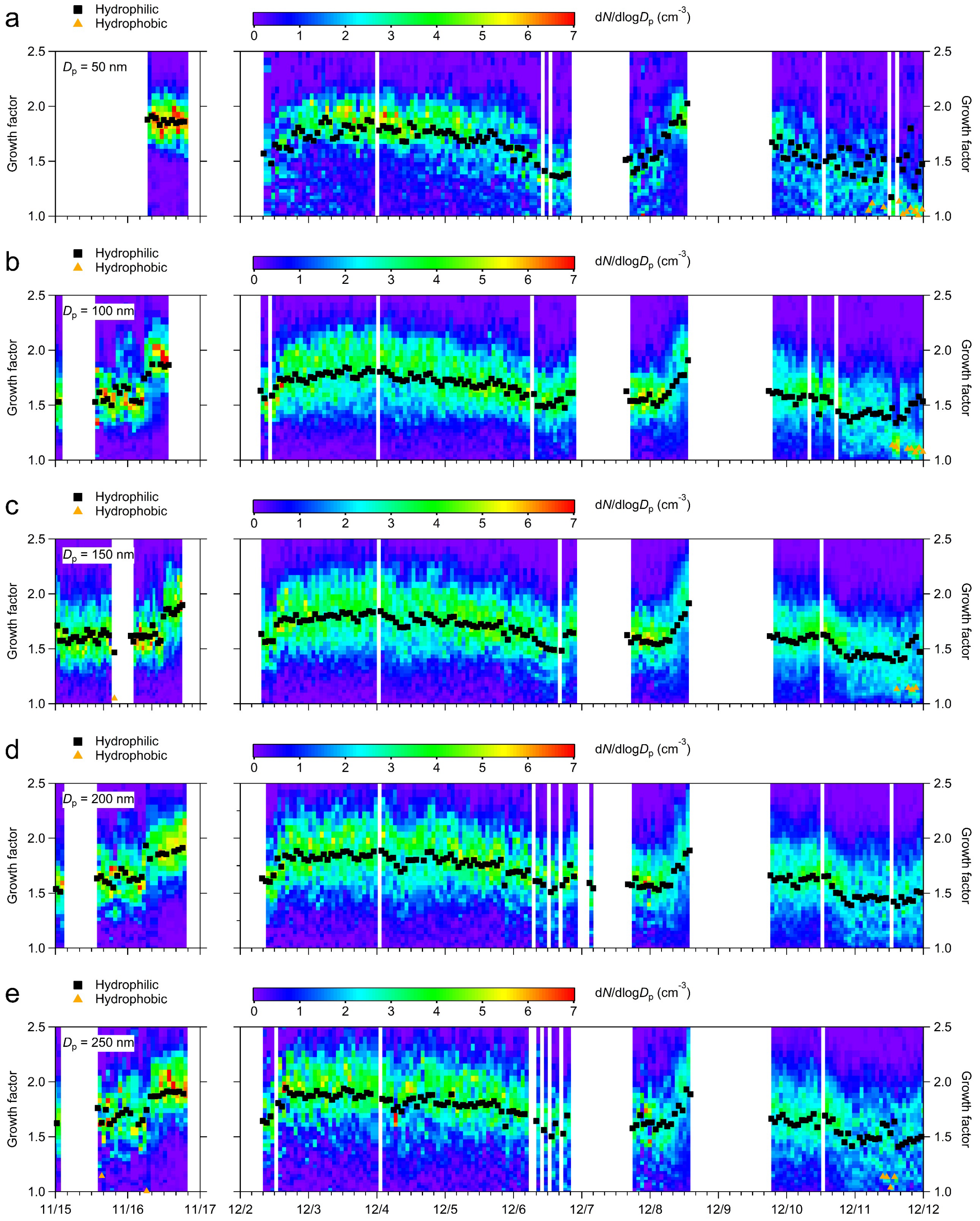

Supplementary Fig. 4 | Particle growth factor measured by the HTDMA. a, Contour plot of particle growth factor (GF) distribution for 50 nm dry particles. The average growth factor of

hydrophilic mode (GF≥1.15) and hydrophobic mode (GF<1.15) are shown in black squares and orange triangles, respectively. **b-e**, Same as plot **a**, but for 100, 150, 200 and 250 nm dry particles.

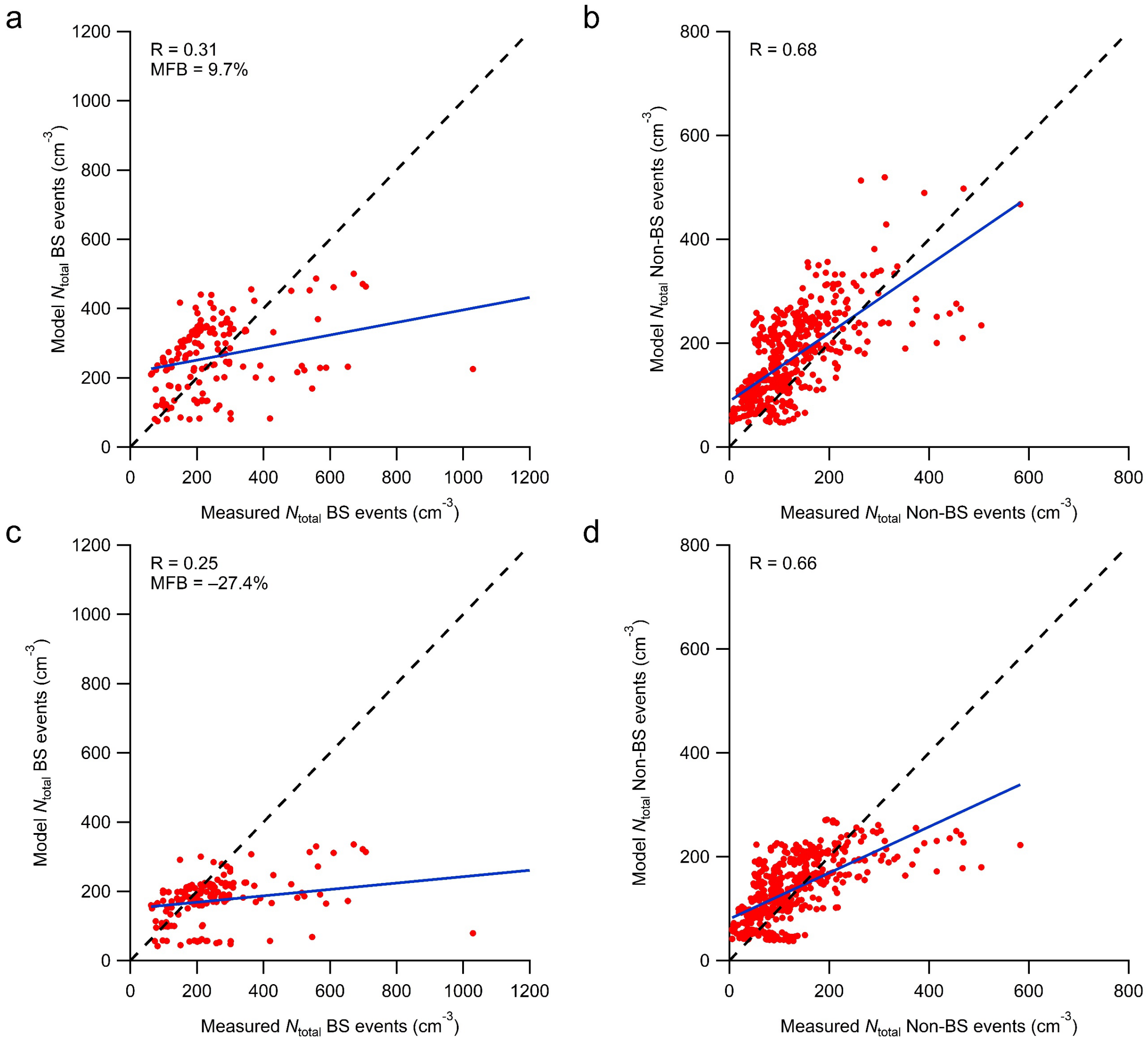

Supplementary Fig. 5 | Comparison between model-simulated and measured particle number concentration. The correlation between model-simulated (NP=5, base simulation) and measured total particle number concentration during blowing snow events in panel **a** and non-blowing snow periods in panel **b**. **c-d**, The same correlation plots for the NP=1 simulation.

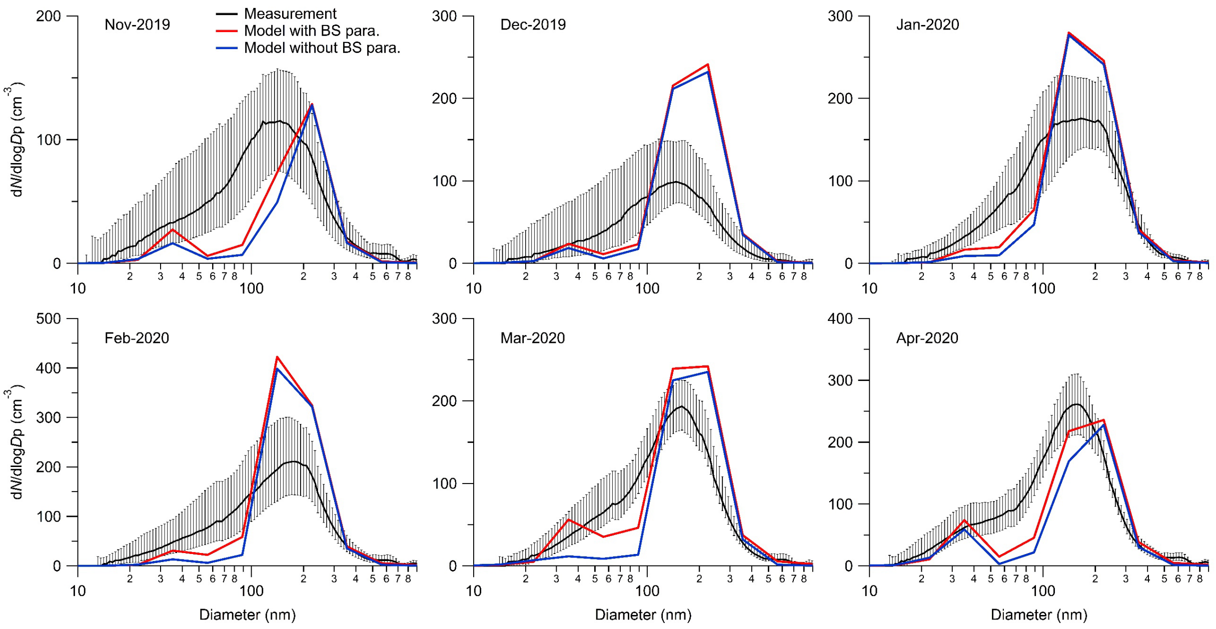

Supplementary Fig. 6 | Comparison between model-simulated (NP=1) and measured particle size distribution. The monthly median values of the measured particle number size distribution are shown in black lines, with error bars showing the 25th to 75th percentiles. The monthly median values of particle number size distribution from NP=1 simulation with and without blowing-snow-produced SSA included are shown in red and blue lines, respectively.

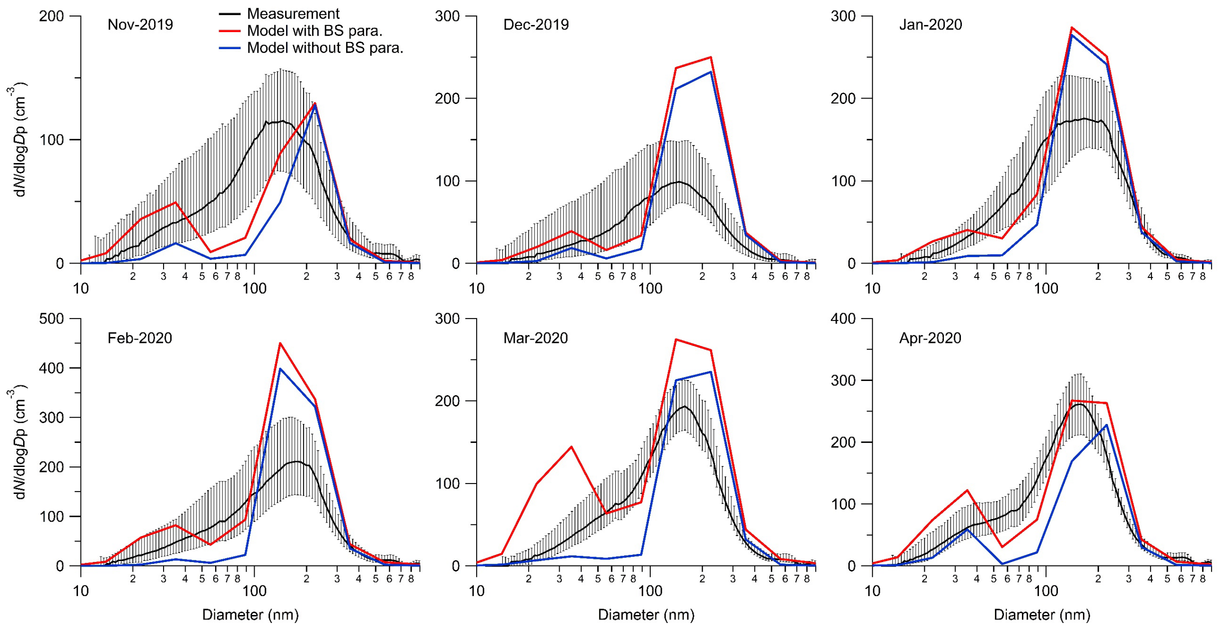

Supplementary Fig. 7 | Comparison between model-simulated (NP=5, low salinity) and measured particle size distribution. The monthly median values of the measured particle number size distribution are shown in black lines, with error bars showing the 25th to 75th percentiles. The monthly median values of particle number size distribution from the low salinity simulation (NP=5) with and without blowing-snow-produced SSA included are shown in red and blue lines, respectively.

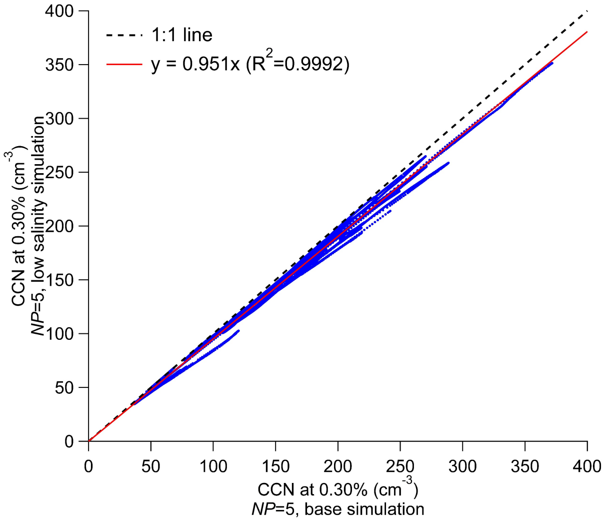

Supplementary Fig. 8 | Comparison of the model-simulated CCN concentrations between the base and low salinity simulations. Total CCN concentration at 0.3% supersaturation from the low salinity simulation as a function of the CCN concentration from the base simulation (blue dots). The dashed black line represents a 1:1 reference line. The red line represents a linear fit with an intercept of 0.

Supplementary References

78. Hasenecz, E. S., Kaluarachchi, C. P., Lee, H. D., Tivanski, A. V & Stone, E. A. Saccharide Transfer to Sea Spray Aerosol Enhanced by Surface Activity, Calcium, and Protein Interactions. *ACS Earth Sp. Chem.* **3**, 2539–2548 (2019).

79. Cochran, R. E. *et al.* Molecular Diversity of Sea Spray Aerosol Particles: Impact of Ocean Biology on Particle Composition and Hygroscopicity. *Chem* **2**, 655–667 (2017).

80. Zieger, P. *et al.* Revising the hygroscopicity of inorganic sea salt particles. *Nat. Commun.* **8**, 15883 (2017).

81. Zelenyuk, A., Cai, Y. & Imre, D. From Agglomerates of Spheres to Irregularly Shaped Particles: Determination of Dynamic Shape Factors from Measurements of Mobility and Vacuum Aerodynamic Diameters. *Aerosol Sci. Technol.* **40**, 197–217 (2006).

82. Gysel, M. *et al.* Closure study between chemical composition and hygroscopic growth of aerosol particles during TORCH2. *Atmos. Chem. Phys.* **7**, 6131–6144 (2007).

83. Nilsson, E. D. *et al.* Turbulent aerosol fluxes over the Arctic Ocean: 2. Wind-driven sources from the sea. *J. Geophys. Res. Atmos.* **106**, 32139–32154 (2001).

84. Stein, A. F. *et al.* NOAA's HYSPLIT Atmospheric Transport and Dispersion Modeling System. *Bull. Am. Meteorol. Soc.* **96**, 2059–2077 (2015).

85. Smith, S. D., Muench, R. D. & Pease, C. H. Polynyas and leads: An overview of physical processes and environment. *J. Geophys. Res. Ocean.* **95**, 9461–9479 (1990).

86. Confer, K. L. *et al.* Impact of Changing Arctic Sea Ice Extent, Sea Ice Age, and Snow Depth on Sea Salt Aerosol From Blowing Snow and the Open Ocean for 1980–2017. *J. Geophys. Res. Atmos.* **128**, e2022JD037667 (2023).
